# Identifying Targets for Interventions to Increase Uptake and Use of Hearing Protection in Noisy Recreational Settings

**DOI:** 10.3390/ijerph18158025

**Published:** 2021-07-29

**Authors:** Michael T. Loughran, Christopher J. Plack, Christopher J. Armitage

**Affiliations:** 1 Manchester Centre for Health Psychology, School of Health Sciences, University of Manchester, Manchester M13 9PL, UK; chris.armitage@manchester.ac.uk; 2 Manchester Centre for Audiology and Deafness, School of Health Sciences, University of Manchester, Manchester M13 9PL, UK; chris.plack@manchester.ac.uk; 3 Department of Psychology, Lancaster University, Lancaster LA1 4YF, UK; 4 Manchester Academic Health Science Centre, Manchester University NHS Foundation Trust, Manchester M13 9QN, UK; 5 NIHR Greater Manchester Patient Safety Translational Research Centre, University of Manchester, Manchester M13 9PL, UK

**Keywords:** hearing prevention, hearing protection interventions, hearing conservation, hearing protection behaviour, behaviour change, recreational noise, recreational noise-induced hearing loss, COM-B

## Abstract

Interventions to increase hearing protection behaviours within noisy recreational settings are limited by the lack of an underpinning evidence base. The aim of the present study was to identify targets for interventions in a population exposed to recreational noise, including those who had used hearing protection (ever-performers) versus those who had not (never-performers). A cross-sectional survey was administered to 185 UK adults who had been involved in noisy recreational activities. Participants had an average age of 36.79 years; the majority were women (68.1%), from a white ethnic background (87.6%), and with non-manual occupations (75.7%). Using Chi-square, MANOVA and ANOVA, we looked for differences in sociodemographic variables and variables from the capabilities, opportunities and motivations model of behaviour change (COM-B) between ever- and never-performers. Ever-performers were more likely to be younger (*p* < 0.050), men (*p* < 0.050), and in a manual occupation (*p* < 0.050) compared to never-performers. Although the two groups felt capable and reported similar opportunities to use hearing protection, never-performers lacked automatic motivation (*p* < 0.001) and reflective motivation (*p* < 0.001) compared to ever-performers. For the first time, the present study identifies potential groups at whom hearing protection interventions might be targeted and what those interventions may contain. Further work is required to develop interventions targeted at older people, women and those in non-manual occupations. Lack of motivation is a key concern, and further work that uses specific theoretical frameworks, such as the PRIME (Plans, Responses, Impulses, Motives, and Evaluations) theory of motivation, may shed light on the kinds of interventions that are needed to boost hearing protection use effectively.

## 1. Introduction

Hearing loss is ranked third in total years lived with a disability, ahead of both diabetes and asthma [1]. Exposure to excess recreational noise (e.g., amplified music, sports-related noise, power tools, but excluding headphones/earphones because mitigation strategies differ) is common [2] and a major contributor to hearing loss and tinnitus [3] that can be mitigated through the use of hearing protection [4,5]. Despite this, it has been estimated that just 2% of the UK adult population always used hearing protection during noisy recreational activities [2]. Moreover, a recent systematic review concluded that the lack of a basic evidence base undermined the effectiveness of interventions aimed at increasing hearing protection use in recreational settings [6]. The purpose of the present study was to gather data to inform the development of behaviour change interventions to promote uptake and use of hearing protection in noisy recreational settings.

Loughran et al.’s [6] systematic review concluded that future hearing protection intervention studies should avoid ad hoc approaches to intervention development and instead adopt systematic approaches, such as the “behaviour change wheel” [7]. For example, in many studies it is not clear how the intervention was developed [8,9,10], meaning that it is impossible to ascertain if the intervention is evidence based and making it difficult to replicate. A key formative stage in intervention design is understanding what needs to change, which can be assessed in terms of the capabilities, opportunities and motivations model of behaviour change (COM-B model [7]). The COM-B model is useful in conceptualising interventions, and Loughran et al.’s [6] systematic review revealed that previous interventions focused exclusively on developing people’s capabilities by increasing knowledge (“psychological capability”) and/or skills (“physical capability”). According to COM-B [7], although addressing knowledge and skills is necessary for behaviour change to occur, it is not sufficient, because behaviour change is additionally driven by physical opportunities (e.g., resources), social opportunities (e.g., social influences), automatic motivation (e.g., impulses) and reflective motivation (e.g., plans).

Thus, a first step in developing hearing protection interventions is to understand what needs to change in terms of people’s capabilities, opportunities and/or motivations. In order to address this question, for the first time, we proactively recruited a group of people who had been exposed to excessive recreational noise over their lifetimes and examined what discriminated those who did use hearing protection (ever-performers) from those who did not (never-performers). Due to ever-performers having already taken the steps to perform hearing protection behaviour, we can hypothesise that they will have significantly greater capabilities, opportunities and motivations than never-performers. It is also important to establish whether any sociodemographic variables (e.g., age, gender, ethnicity, education, and socioeconomic status) differ within these naturally occurring hearing protection groups in order to help identify groups to target in future interventions. Based upon recent epidemiological data from the UK [2], we can hypothesise that, compared to never-performers, ever-performers will be younger and men.

## 2. Materials and Methods

Ethical approval was granted by the Division of Psychology and Mental Health, University of Manchester, June 2018. Reference: 2018-3556-6133.

### 2.1. Design, Recruitment and Hearing Protection Groups

A cross-sectional survey was made available from June 2018 until June 2019 to any UK-based individual aged 18–69 years. The final dataset included anyone able to confirm their involvement in at least one noisy recreational activity (e.g., amplified music events, indoor and outdoor sports, use of power tools), defined by vocal exertion in presence of background noise (>85 dBA [11,12]). Recruitment was through social media advertising in order to capture a large demographic exposed to recreational noise; other methods included university promotion through email, websites and posters. There was no monetary reward for those who completed the survey. One hundred and eighty-five people provided usable data.

Those included had the option to say whether they had performed any hearing protection behaviours (ever-performers; e.g., earplugs, earmuffs, other techniques) while taking part in noisy recreational activities, or not (never-performers). Originally, the participants were asked, “Do you use hearing protection when you do this activity?” [12], to which they could answer on a Likert-type scale with the options “always, often, sometimes, seldom, never” [13]. These results were then dichotomously coded as ever-performers (use at least some of the time) and never-performers (no use) for analysis proposes, and for comparison to previous epidemiological studies [2].

### 2.2. Measures

#### 2.2.1. Sociodemographic

Sociodemographic measures included age, gender, ethnicity and socioeconomic status. According to the UK Office of National Statistics, ethnicity was divided into White versus Black, Asian, or Minority Ethic (BAME), and socioeconomic status was split into manual versus non-manual work [14].

#### 2.2.2. Psychosocial

Keyworth et al.’s [15] validated measure was adapted to assess people’s capabilities, opportunities and motivations to engage in hearing protection. The scale comprises six COM-B statements that are rated on 0–10-point Likert-type scales, anchored with “strongly disagree” and “strongly agree”. *Psychological capability* captured if the person felt they had the knowledge and skills to use hearing protection, “*I have the knowledge and skills, and I have the ability to remember, pay attention and make decisions to use hearing protection*”. *Physical capability* explored if they felt they had the physical skills and strength to perform the behaviour, “*I have enough physical stamina and I have sufficient physical skills to use hearing protection*”. *Physical opportunity* assessed people’s perceptions of having the necessary resources and reminders to use hearing protection, “*I have sufficient time, the necessary resources, and the reminders to use hearing protection*”. *Social opportunity* considered if they felt they had adequate social support, “*I have the necessary support from people (e.g., from friends and family) to use hearing protection*”. *Reflective motivation* evaluated if the person had the desire and wanted to use hearing protection, “*I have the desire to and I want to use hearing protection*”. *Automatic motivation* assessed if the behaviour was performed without realisation, “*Using hearing protection is something I do before I realise I’m doing it*”.

### 2.3. Data Analysis

Statistical analyses were conducted using SPSS version 23. Chi-squared and univariate analyses of variance (ANOVAs) were used to explore differences between the current sample of adults exposed to recreational noise and a UK population study for representativeness [2]. Chi-squared and one-way multivariate analysis of variance (MANOVA) and ANOVA tests were used to explore potential differences between ever-performers and never-performers of hearing protection in terms of their sociodemographic variables and their capabilities, opportunities and motivations to use hearing protection.

## 3. Results

### 3.1. Participant Characteristics

Of the 185 participants recruited, most were women (68.1%, *n* = 126), with a white ethnic background (87.6%, *n* = 162), a higher education (93.5%, *n* = 173), non-manual occupation (75.7%, *n* = 140), and with an average age of 36.79 years (SD = 15.328). Compared with a nationally representative sample [2], the present sample was almost identical in rates of hearing protection used (ever-performers = 23.7%; never-performers = 76.3%; χ^2^ (1, *N* = 7775) = 0.94, *p* = 0.333) by adults exposed to recreational noise. However, they were younger (F(1, 10,584) = 3287.007, *p* < 0.050), more likely to be women (χ^2^ (1, *N* = 10,495) = 21.82, *p* < 0.050), more likely to report a white ethnic background (χ^2^ (1, *N* = 10,582) = 5.85, *p* < 0.050) and more likely to be in a non-manual occupation (χ^2^ (1, *N* = 10,599) = 108.10, *p* < 0.050).

Comparable with nationally representative data [2], among adults exposed to recreational noise, the number of never-performers (76.3%, *n* = 141) exceeded ever-performers (23.7%, *n* = 44), and with only 4.3% (*n* = 8) “always” using hearing protection, 2.7% (*n* = 5) “often”, 8.6% (*n* = 16) “sometimes”, and 8.1% (*n* = 15) “seldom”, there is still work to be done to promote hearing protective behaviours. Consistent with our hypothesis, comparisons revealed that ever-performers were younger (M = 32.18, SD = 13.003; F(1, 183) = 5.348, *p* < 0.050), and more likely to be men (45.5% versus never-performers = 26.2%; χ^2^ (1, *N* = 183) = 6.19, *p* < 0.050) and in a manual occupation (11.4% versus never-performers = 2.1%; χ^2^ (1, *N* = 148) = 5.69, *p* < 0.050). No differences between hearing protection groups were found for ethnicity (χ^2^ (1, *N* = 181) = 0.055, *p* = 0.814), nor education (χ^2^ (1, *N* = 182) = 2.27, *p* = 0.132) (see Table 1).

### 3.2. Capabilities, Opportunities and Motivations to Perform Hearing Protection Behaviour

Using Pillai’s trace, MANOVA indicated that there were significant differences between ever-performers and never-performers of hearing protection in terms of their capabilities, opportunities and motivations (V = 0.150, F(6, 178) = 5.223, *p* < 0.001). ANOVAs revealed significant differences between groups for reflective motivation (“never” M = 4.80, SD = 3.258, versus “ever” M = 7.34, SD = 2.667; F(1, 183) = 22.081, *p* < 0.001), and automatic motivation (“never” M = 1.44, SD = 2.297, versus “ever” M = 3.36, SD = 3.335; F(1, 183) = 18.667, *p* < 0.001) (see Table 2). The results indicate that never-performers are lacking the automatic and reflective motivation to perform the behaviour, compared to ever-performers. Furthermore, there is still scope to improve ever-performers’ automatic and reflective motivation.

No statistically significant differences were found between groups in terms of their social (“never” M = 4.41, SD = 3.364, versus “ever” M = 5.45, SD = 3.358; F(1, 183) = 3.228, *p* = 0.074) and physical (“never” M = 4.87, SD = 3.442, versus “ever” M = 5.82, SD = 2.847; F(1, 183) = 2.777, *p* = 0.097) opportunities. However, although results indicate similar opportunities, with scores close to the midpoint it would suggest that there is quite a large scope available to improve physical and social opportunities for both. There were also no differences found for psychological (“never” M = 8.66, SD = 2.277, versus “ever” M = 8.61, SD = 2.223; F(1, 183) = 0.014, *p* = 0.907) and physical (‘“never” M = 8.98, SD = 2.065, versus “ever” M = 9.14, SD = 1.488; F(1, 183) = 0.220, *p* = 0.639) capabilities, and it appears that both groups feel they have sufficient capabilities.

## 4. Discussion

The major finding of this study is that, with similar rates of capability (psychological and physical) and opportunity (social and physical) recorded across both groups, it is ever-performers’ greater reflective (e.g., plan to use earplugs) and automatic motivation (e.g., impulse to use earplugs) driving their use of hearing protection. Use of the COM-B model has highlighted never-performers’ lack of reflective and automatic motivation as a concern, and potential targets of the content addressing what needs to change to increase use, and uptake of hearing protection. In order to address this gap, further research is required to pinpoint what is energising these motivational differences, and implementation of theoretical frameworks such as the PRIME (Plans, Responses, Impulses, Motives, and Evaluations) theory of motivation [16] could aid future intervention development, as seen with smoking cessation [17]. PRIME provides a comprehensive overview of motivation within a single theory [18,19], and suggests that behaviour can only be influenced if there is first sufficient desire (reflective motivation), which can then action the impulse (automatic motivation) [20]. The application of qualitative research grounded by theoretical models and frameworks may help address these motivational gaps further.

Comparable with Armitage et al.’s [2] research using a nationally representative sample, those using hearing protection were more likely to be men, and younger than never-performers. Ever-performers were more likely to be in manual occupations, potentially owing to greater familiarisation with hearing protection due to mandatory UK health and safety protocols [21]. These findings are similar to those found in Australia [22], where the use of hearing protection at work was a significant predictor of recreational hearing protection use. The implications of these findings are that older people, women, and those who work in non-manual occupations are potential groups to target for future interventions to try to increase use, and uptake, of hearing protection.

Other potential avenues for change may have presented themselves through the low scoring recorded for physical and social opportunities. In order to address these gaps, further explorative research is required, potentially achieved through qualitative fact-finding informed by the COM-B model itself, similar to that seen within the fields of dietary patterns [23,24] and cancer research [25]. Such research could bolster the current evidence base for future interventions. We currently know that within the UK, physical opportunities (e.g., resources) such as hearing protection devices are not required to be available during noisy recreational activities for attendees [21], and that social opportunities (e.g., social norms; peer behaviour and opinions) are associated with having a negative influence on hearing protection behaviour [26]. However, we do not know what physical (e.g., resources/reminders) and social (social influences e.g., friends, peers, family) opportunities ever- and never-performers have had afforded to them that may positively affect their behaviour.

### 4.1. Strengths

First, this study is the first of its kind to assess the capabilities, opportunities and motivations of ever- and never-performers of hearing protection during noisy recreational activities, highlighting potential targets to drive improved behaviour, and although these measures were self-reported, the tool is known to be reliable and valid [15]. Second, the present sample is reasonably representative in comparison with a previous population study [2] with regard to numbers of recreationally noise-exposed ever- and never-performers of hearing protection behaviour.

### 4.2. Limitations

First, although the present sample was representative in terms of the numbers of UK adults known to use hearing protection recreationally [2], it was unrepresentative in some important respects (e.g., in the distribution of age, gender, ethnicity, and socioeconomic status), and it would be valuable to replicate the present findings with a more representative sample. Second, hearing protection behaviours were recorded through self-report rather than assessed objectively. This may have led to over- or under-estimation in some instances, and it would be beneficial in future to assess these factors through the use of technology or observations. Third, we considered hearing protection behaviour across multiple recreational activities, which could be viewed as being problematic as noise levels will vary. However, with noise levels defined by vocal exertion known to be reliable [27], and only those greater than 85 dBA included, then any activity exceeding this level warrants protective measures [21].

## 5. Conclusions

Interventions addressing reflective and automatic motivation, and targeting older people, women, and those in non-manual occupations, may have the potential to increase hearing protection behaviour during noisy recreational settings. Further research is required to explore the motivational differences between ever- and never-performers using theoretical frameworks, such as the PRIME theory of motivation, with the prospect of developing an intervention. Additionally, it would be valuable to replicate the findings in a larger more representative sample.

## Figures and Tables

**Table 1:** Sociodemographic comparisons between hearing protection groups.

Variable	Never-Performers (*n* = 141)			Ever-Performers (*n* = 44)			Comparisons
	%	M	SD	%	M	SD	*p*
Gender	--	--	--	--	--	--	0.013 *
Women	73	--	--	52.3	--	--	--
Men	26.2	--	--	45.5	--	--	--
PNTS	0.7	--	--	2.3	--	--	--
Age	--	38.23	15.751	--	32.18	13.003	0.022 *
Ethnicity	--	--	--	--	--	--	0.814
White	88	--	--	86.4	--	--	--
BAME	10.6	--	--	9.1	--	--	--
DNS	1.4	--	--	4.5	--	--	--
Education	--	--	--	--	--	--	0.132
Higher	95	--	--	88.6	--	--	--
Lower	3.5	--	--	9.1	--	--	--
DNS	1.4	--	--	2.3	--	--	--
Socioeconomic status	--	--	--	--	--	--	0.017 *
Non-manual	75.2	--	--	77.3	--	--	--
Manual	2.1	--	--	11.4	--	--	--
DNS	22.7	--	--	11.4	--	--	--

* = *p* < 0.050; PNTS—Prefer not to say; DNS—Did not say.

**Table 2:** Differences between groups for capabilities, opportunities and motivations to use hearing protection.

Variable	Never-Performers (*n* = 141)			Ever-Performers (*n* = 44)			Univariate ANOVA			
	%	M	SD	%	M	SD	df	df E	F	*p*
Psychological capability	--	8.66	2.277	--	8.61	2.223	1	183	0.014	0.907
Physical capability	--	8.98	2.065	--	9.14	1.488	1	183	0.022	0.639
Social opportunity	--	4.41	3.364	--	5.45	3.358	1	183	3.228	0.074
Physical opportunity	--	4.87	3.442	--	5.82	2.847	1	183	2.777	0.097
Reflective motivation	--	4.80	3.258	--	7.34	2.667	1	183	22.081	<0.001 *
Automatic motivation	--	1.44	2.297	--	3.36	3.335	1	183	18.667	<0.001 *

* = *p* value < 0.050.

## Data Availability

The datasets used and/or analysed during the current study are available from the corresponding author on reasonable request.
